# Association between pregnant women with rheumatoid arthritis and preeclampsia: A systematic review and meta-analysis

**DOI:** 10.1097/MD.0000000000034131

**Published:** 2023-06-30

**Authors:** Lv Tian, Zhiyuan Zhang, Yuting Mao, Minru Zong

**Affiliations:** a School of Nursing, Jilin University, Changchun, China; b Affiliated Stomatological Hospital of Zunyi Medical University, Zunyi Medical University, Zunyi, China; c Department of Rehabilitation, The Third Hospital of Jilin University, Changchun, China.

**Keywords:** maternal morbidity, meta-analysis, preeclampsia, RA, systematic review

## Abstract

To examine the association between pregnant women with rheumatoid arthritis (RA) and the risk of preeclampsia.

**Methods:**

This study was registered on the International Prospective Register of Systematic Reviews (PROSPERO) under the number CRD42022361571. The primary outcome was preeclampsia. Two evaluators independently reviewed the included studies, assessed their risk of bias, and extracted the data. Unadjusted and adjusted ratios with 95% confidence intervals and 95% prediction intervals were calculated. Heterogeneity was quantified using the *І*^2^ statistic, where *І*^2^ ≥ 50% indicated the presence of significant heterogeneity. Subgroup and sensitivity analyses were performed to test the robustness of the overall findings.

**Results:**

A total of 8 studies, including 10,951,184 pregnant women, of whom 13,333 were diagnosed with RA, met the inclusion criteria. Meta-analysis revealed that pregnant women with RA were significantly more likely to develop preeclampsia than those without RA (pooled odds ratio, 1.66; 95% confidence interval, 1.52–1.80; *P* < .001; *І*^2^ < .001).

**Conclusion:**

RA during pregnancy is associated with higher odds of preeclampsia.

## 1. Introduction

Rheumatoid arthritis (RA) is defined as a systemic autoimmune lesion associated with a chronic inflammatory process.^[1]^ RA is a multifactorial disease caused by genetic, environmental, and stochastic factors.^[2]^ Over the past 30 years, many scientists have extensively studied the changes in the prevalence and incidence of RA. These studies have shown that RA is a global disease distributed worldwide regardless of race, sex, ethnicity, nationality, age, etc. However, prevalence and incidence measurements vary according to population characteristics and change over time.^[3]^ Although the mechanisms of RA have not been fully elucidated, studies have reported the involvement of cytokines such as tumor necrosis factor-α (TNF-α) and interleukin 6 in the pathophysiological processes of RA.^[4]^

Preeclampsia is a multisystem syndrome that complicates approximately 5% of pregnancies and is one of the leading causes of maternal mortality worldwide, accounting for approximately 14% of all maternal deaths.^[5]^ In 2018, geographic, social, economic, and ethnic differences could explain the different prevalences of preeclampsia seen in different populations. Worldwide, preeclampsia is the second leading cause of maternal death, with estimates of at least 16% in low- and middle-income countries and >25% in some countries in Latin America.^[6]^ End-organ disorders caused by preeclampsia are diverse and may include proteinuria, acute kidney injury, liver dysfunction, hemolysis, thrombocytopenia, and, less commonly, liver rupture, seizures (eclampsia), stroke, and death. There are many risk factors for the development of preeclampsia, such as a history of preeclampsia in previous pregnancies, diabetes mellitus, hypertension, obesity, and multiple pregnancies.^[7]^ In addition, women with preeclampsia are at greater risk for cardiovascular disease later in life.^[8–10]^ Although the etiology of preeclampsia is unknown, it is currently believed that placental abnormalities leading to placental malperfusion and dysfunction, syncytial trophoblast stress, oxidative stress, imbalance of circulating placental angiogenic factors, perturbation of the renin-angiotensin system (RAS), placental senescence inflammation, endothelial dysfunction and influenced by maternal genetics, epigenetics, immune abnormalities, lifestyle, and environmental factors are associated with the pathophysiology of this disorder.^[11–21]^

As mentioned above, maternal RA potentially impacts preeclampsia, but the association between the 2 is controversial. Five cohort studies^[22–26]^ of 10,690,212 participants found a significant association between maternal RA and increased risk of preeclampsia, compared with 3 studies^[27–29]^ showing no statistically significant association between maternal RA and preeclampsia.

In addition, the association between maternal rheumatoid and the risk of preeclampsia has received less attention. This topic is relevant to public health and clinical practice. Therefore, we conducted a systematic evaluation with the primary aim of compiling and critically assessing the available evidence on the association between maternal RA and the risk of preeclampsia using formal systematic evaluation and meta-analysis techniques.

## 2. Methods

### 2.1. Ethical approval

This meta-analysis is based entirely on previously published studies that have declared ethical approval and did not collect or utilize the original clinical raw data; therefore, ethical approval was not required for this study.

### 2.2. Registration information

This meta-analysis was reported according to the Preferred Reporting Items for Systematic Reviews and Meta-Analyses (PRISMA). And it was registered on the International Prospective Register of Systematic Reviews (PROSPERO). The number was CRD42022361571.

### 2.3. Search strategy

The search strategy was designed to include studies published in the English language only. A comprehensive search strategy was conducted for English-language articles examining pregnant women with RA and preeclampsia. The literature was searched from inception to September 2022 in Scopus, Web of Science, PubMed, Cochrane Library, and Embase databases. The following terms were used: (Arthritis OR Rheumatoid Arthritis OR Rheumatoid OR (rheuma* OR arthrit* OR polyarthrit*)) AND (Preeclampsia OR Pre-Eclampsia OR Pregnancy-induced hypertension OR Hypertensive disorders of pregnancy OR Gestational hypertension OR Pregnancy-associated hypertension OR Pregnancy toxemia OR pregnancy hypertension OR EPH-gestosis) AND (Maternal OR Pregnancy). The terms were adjusted according to each database’s syntax and indexing system.

### 2.4. Inclusion and exclusion criteria of literature

The inclusion criteria developed for screening eligible publications were as follows: The type of study was a case-control study or cohort study; the outcome of interest was the risk in preeclampsia; estimates such as odds ratios (ORs) or relative risks (RRs) with the corresponding 95% confidence intervals (CIs) were reported, or sufficient data were provided to perform the calculation. Exclusion criteria were as follows: the study was not in humans, for example, in vitro and in vivo studies or animal studies; the article was in the review category; risk estimates could not be calculated; duplication; the destination data cannot be obtained; non-English articles.

### 2.5. Data extraction and quality assessment

Preeclampsia was the only outcome evaluated in this systematic review and meta-analysis. Diagnostic criteria for preeclampsia were based on the guidelines established by the American Congress of Obstetricians and Gynecologists. Preeclampsia was defined as new-onset hypertension (systolic blood pressure ≥ 140 mm Hg or diastolic blood pressure ≥ 90 mm Hg) after 20 weeks of gestation, accompanied by proteinuria (≥300 mg/24 hours) or other signs of end-organ dysfunction.^[7]^

Two participants extracted the data independently according to a predefined data form and then assessed the quality of the articles. If disagreements were encountered, discussions were held to reach an agreement, or a third person was consulted regarding recommendations. Data extracted included authors’ names, year, region, study design, comparisons, sample size, OR or RR with 95% CI, quality and adjustment factors, assessment method, and the age of the participants.

The quality of observational studies was assessed by the Newcastle-Ottawa scale, which has 3 columns: selection, comparability, and outcome/exposure. The total score is 9 stars, with 6 or more stars for high-quality literature and 4 to 5 stars for moderate quality.^[30]^

### 2.6. Statistical analysis

The association between pregnant women with RA and preeclampsia was assessed by pooling the ORs with the corresponding 95% CIs. Since the absolute risk of preeclampsia is relatively low, the RRs were expected to yield similar estimates as ORs and thus were all pooled as ORs. When multiple ORs were provided in a single study, the one that controlled the most confounders was selected. Heterogeneity between studies was tested by Q-test at the *P* = .1 level and then based on *I*^2^ statistics: *I*^2^ < 50% of respondents indicated no significant statistical heterogeneity and therefore a fixed-effects model was selected, *I*^2^ > 50% indicated statistical heterogeneity,^[31,32]^ and a random effects model was used. Sensitivity analyses were achieved by the leave-one-out method. Publication bias was assessed by observing whether the funnel plot was symmetrical and by calculating Begg and Egger test values.^[33]^ Data were processed using the statistical software Stata 15.0 (Stata Corp., College Station, TX). *P* values < 0.05 were considered statistically significant.

## 3. Results

### 3.1. Literature search and research characteristics

**Figure 1.**
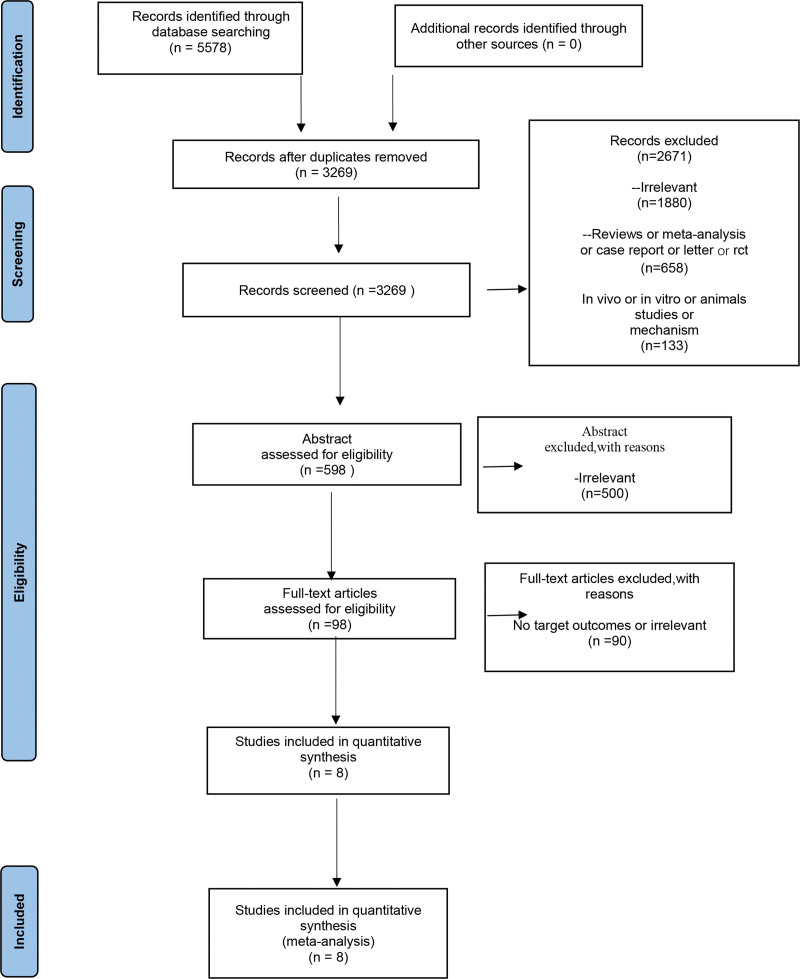
Preferred Reporting Items for Systematic Reviews and Meta-Analysis (PRISMA) flow chart.

Figure 1 shows the process of literature search and selection of studies. The search yielded 5578 records, and the elimination of duplicates yielded 2309 articles. After reading the titles and abstracts, 98 articles were retained. Finally, the full text was read through to filter the literature. Of these, 90 were excluded, mainly due to a lack of data on the association between RA and preeclampsia. A total of 8 studies met the inclusion criteria. The meta-analysis included 8 articles involving a total of 10,951,184 participants, published between 2006 and 2018. Four of the studies^[22,23,27,28]^ were conducted in North America, 3 in Europe,^[24,26,29]^ and 1 in Asia.^[25]^ Six studies were adjusted for confounders, and all included studies were of high or moderate quality. Table 1 presents more detailed information on the included studies.

**Table 1:** Characteristics of individual studies included in the meta-analysis.

Author (year)	OR (95% CI)	RR (95% CI)	Study design	Comparison	Region
Herng-Ching 2009	2.22 (1.59–3.11)		Cohort	Women with RA vs women without RA	Asia (China,Taiwa)
Hissah Aljary 2018	1.71 (1.52–1.89)		Cohort	Women with RA vs women without RA	North America (USA)
K T Jørgensen 2009		1.42 (1.08–1.84)	Cohort	RA with Person-years	Europe (Denmark)
M. Nørgaard 2010	1.5 (1.15–1.94)		Cohort	Women with RA vs women without RA	Europe (Denmark and Switzerland)
Gretchen Bandoli 2017	1.62 (1.04–2.53)		Cohort	Women with RA vs women without RA	North America (USA, Canada)
Marianne Wallenius 2013	1.4 (1–1.9)		Cohort	Women with RA vs With Reference deliveries	Europe (Norway)
Cheryl Barnabe 2011	2.33 (0.76–7.14)		Cohort	Women with RA vs With RA controls	North America (Canada)
Susan D. Reed 2006	1.55 (0.97–2.5)		Cohort	Women with RA vs women without RA	North America (USA)
Author (year)	Incident and sample size	Age of the study participators	Assessment method	Quality	Adjustment factors
Herng-Ching 2009	(51) 11,472	No age limit	RA: ICD-9	High	Adjusted *
Hissah Aljary 2018	(413) 8417,607	No age limit	RA: ICD-9	Moderate	Adjusted †
K T Jørgensen 2009	(57) 1387,186	Age limit (15–49)	RA: ICD-8	High	Adjusted ‡
M. Nørgaard2010	(60) 871,579	No age limit	RA: ICD-8, 9, 10	Moderate	Unadjusted
Gretchen Bandoli 2017	(42) 2368	No age limit	RA: Mother to Baby database	High	Adjusted§
Marianne Wallenius 2013	(45) 257,942	No age limit	RA: ICD-10	High	Adjusted∥
Cheryl Barnabe 2011	(7) 188	Age limit (21–42)	RA: ICD-9	High	Unadjusted
Susan D. Reed 2006	(19) 2842	No age limit	RA: ICD-9	High	Adjusted¶

CI = confidence interval, ICD-8 = International Classification of Diseases, ICD-9 (ICD-9-CM) = International Classification of Diseases, Ninth Revision, Clinical Modification, ICD-10 (ICD-10-CA) = International Classification of Diseases, Tenth Revision, Canadian Enhancement, Incident = incident (RA and preeclampsia), OR = odds ratio, RA = rheumatoid arthritis, RR = relative risk.

* Adjusted for marital status, infant gender, parental age difference, and monthly family income.

† Adjusted for age, race, income, and type of insurance.

‡ RRs are adjusted for age, birth cohort, calendar period, marital status, number of children and age at birth of first child.

§ Propsensity scores for adjustment include race/ethnicity, socioeconomic status, medical comorbidities, pregnancy smoking, pre-pregnancy BMI, maternal age, and gestational age at enrollment.

∥ Adjusted for maternal age at delivery and smoking at time of conception.

¶ Adjusted for maternal age, smoking, and delivery year, relative risk estimated from odds ratio utilizing general linear models for common outcomes.

### 3.2. Overall meta-analysis

**Figure 2.**
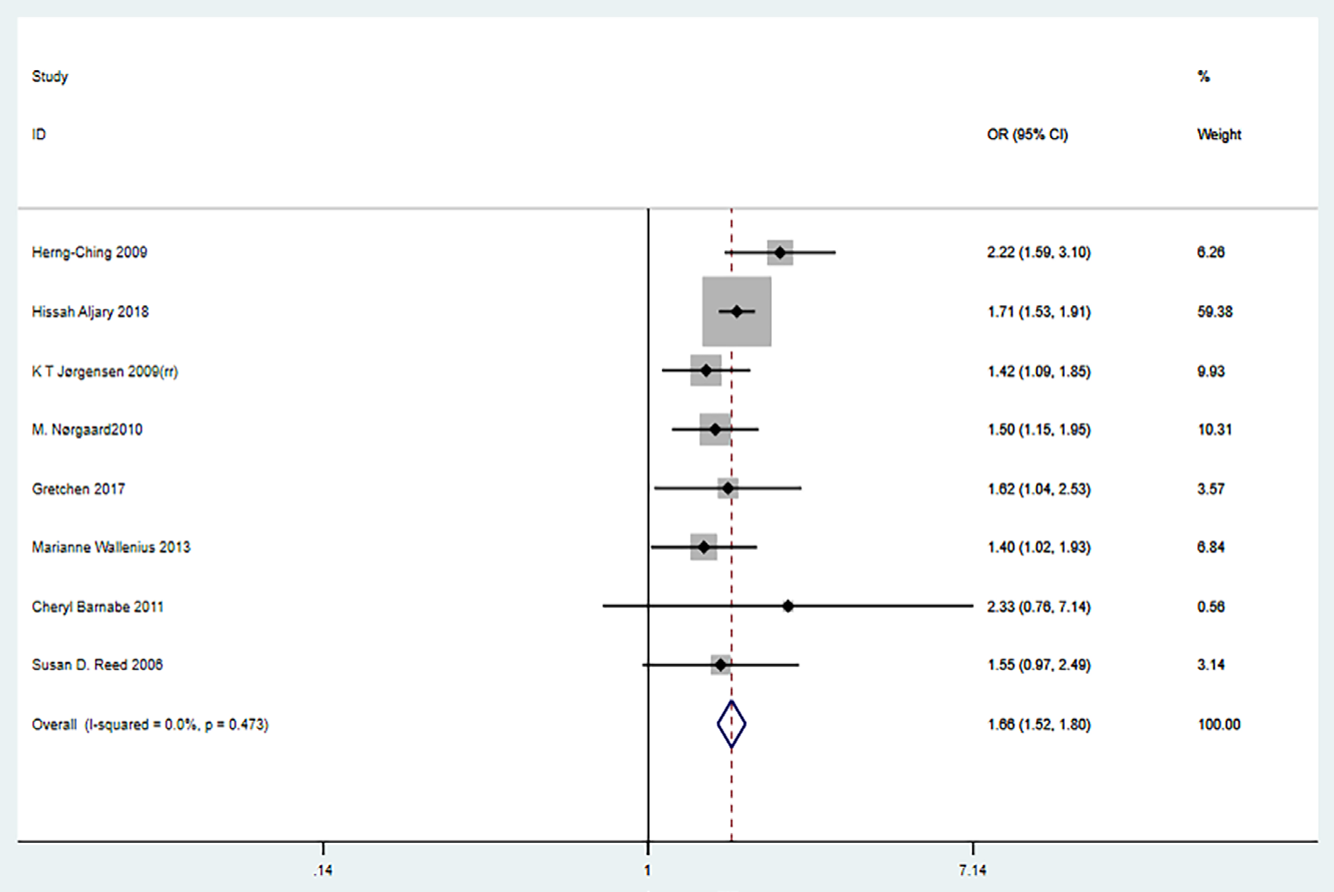
Forest plot of the association between pregnant women with rheumatoid arthritis and preeclampsia.

Eight articles examining the association between maternal RA and the risk of preeclampsia were included in the overall meta-analysis. No significant heterogeneity was observed, and combined effect estimates with a fixed-effects model were shown in a forest sample. Pooled results showed that maternal RA and preeclampsia were statistically significantly associated (OR = 1.66, 95% CI = 1.52–1.80, *P* < .001; Fig. 2).

### 3.3. Subgroup analysis

The results of subgroup analyses of the association between maternal RA and preeclampsia are shown in Table 2. Overall, the direction and magnitude of the effect of maternal RA on preeclampsia were consistent across most of the prespecified subgroup analyses. Pregnant women with RA had a significantly increased probability of preeclampsia.

**Table 2:** Summary of pooled ORs with CI in the meta-analysis.

			Heterogeneity		Significant		
Analysis	No.of studies	OR (95% CI)	*P*	*I*^2^ (%)	*Z*	*P*	M*
Overall	8	1.66 (1.52,1.80)	.473	0.001	11.77	.001	R†
Country							
Asia	1	2.22 (1.59–3.11)	NA	NA	NA	NA	NA
North America	4	1.70 (1.54–1.89)	.92	0.001	10.13	.001	R†
Europe	3	1.45 (1.23–1.70)	.84	0.001	4.47	.001	R†
Adjustment factors							
Adjusted	6	1.67 (1.53–1.83)	.34	11.4%	11.31	.001	R†
Unadjusted	2	1.54 (1.19–1.98)	.45	0.001	3.30	.001	R†
Quality							
High	6	1.61 (1.38–1.87)	.35	9.9%	6.09	.001	R†
Moderate	2	1.68 (1.52–1.86)	.37	0.001	10.08	.001	R†
Age of the study subjects							
No limit	6	1.68 (1.54–1.84)	.43	0.001	11.46	.001	R†
Limit	2	1.46 (1.13–1.89)	.40	0.001	2.85	.004	R†
Sample size							
A	5	1.71 (1.42–2.05)	.36	7.7%	5.62	.001	R†
B	3	1.64 (1.50–1.81)	.34	6.3%	10.34	.001	R†

A = >1 million, B = <1 million, CI = confidence interval, OR = odds ratio, NA = not applicable.

* Model of meta-analysis.

†Fixed effects model.

‡Random effects model.

### 3.4. Sensitivity analysis and publication bias

**Figure 3.**
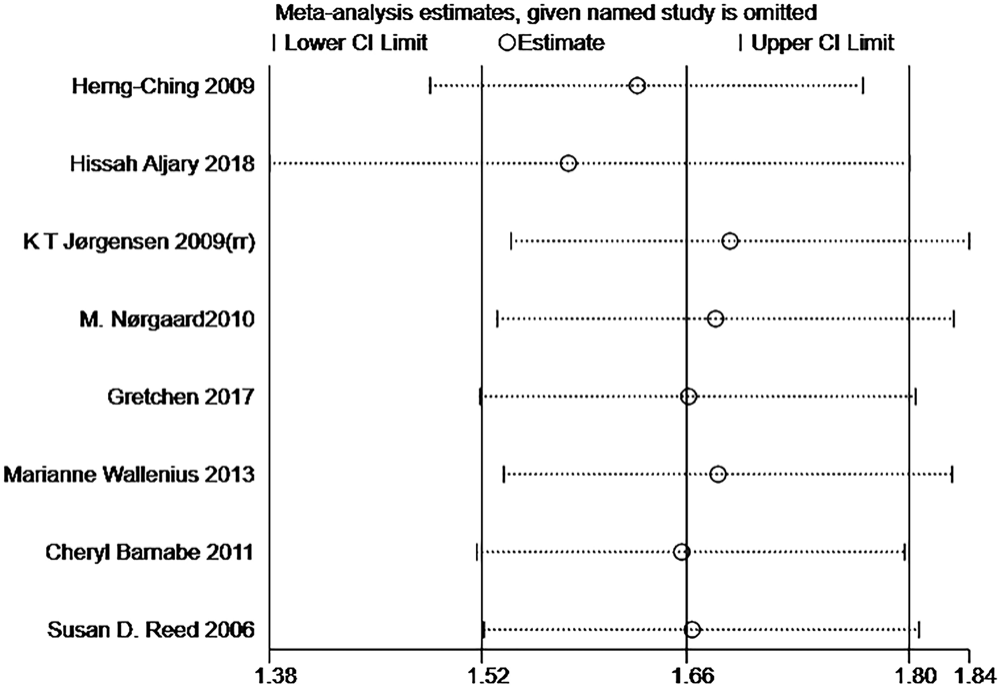
Sensitivity analyses.

**Figure 4.**
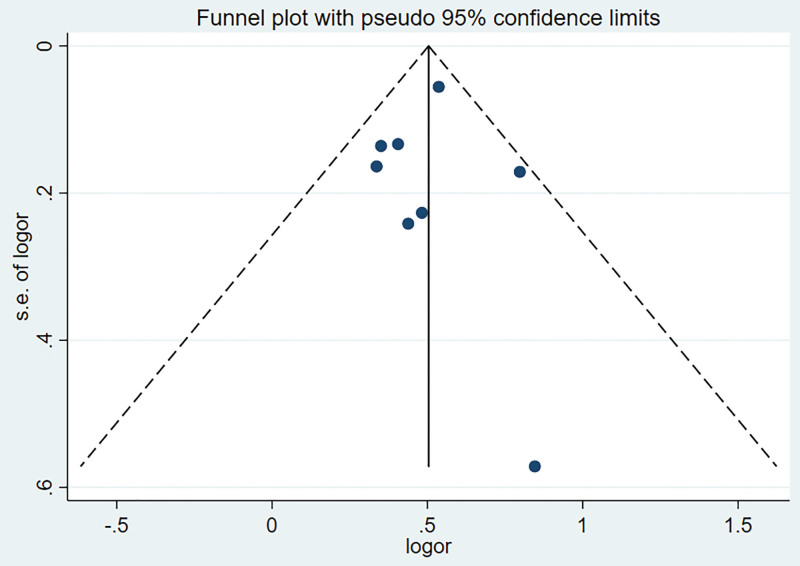
Funnel plot (potential publication bias of included studies).

Sensitivity analysis was performed using the leave-one-out method with stable results (Fig. 3). The funnel plot shape was symmetrically distributed, and Begg test and Egger test showed no publication bias (Begg test: *Z* = 0.37; *P* = .711; Egger test: *t* = −0.22; *P* = .835; (Fig. 4).

## 4. Discussion

This systematic evaluation showed that women with RA in pregnancy were significantly more likely to develop preeclampsia than women without RA in pregnancy. This association was very consistent across all predefined subgroups (region, study quality, age of participants, sample size, and adjusting factors). The effect of preeclampsia in women with RA during pregnancy did not change after sensitivity analysis, which indicates that the results are robust.

Although the underlying mechanisms between pregnant women with RA and preeclampsia are not clear, several mechanisms may explain how RA during pregnancy is involved in the pathogenesis of preeclampsia. Immunoinflammatory disorders (RA) may be associated with ACE/ACE2 imbalance.^[34]^ The ACE2 receptor is an important component of the RAS, which converts angiotensin II to angiotensin 1 to 7. The RAS is an important regulator of placental function because of its role in controlling trophoblast proliferation, angiogenesis, and blood flow. The RAS significantly regulates uteroplacental blood flow through the balance of its vasoconstrictor and vasodilator pathways.^[35]^ Alterations in the RAS system may play a role in the pathophysiology of preeclampsia. RA is usually characterized by an insidious onset of symptoms, but the disease progresses and gets progressively worse over time. Synovial macrophages release cytokines such as tumor necrosis factor-alpha (TNF-α), interleukin-1 (IL-1), and interleukin 6.^[36]^ TNF-α is a multifunctional Th1 cytokine and one of the most important inflammatory cytokines. During pregnancy, TNF-α affects hormone synthesis, placental structure, and embryonic development,^[37]^ and in vitro studies have shown that TNF-α on placental syncytial trophoblasts activates maternal monocytes bound to LFA-1 and induces apoptosis.^[38]^ It has also been shown that TNF-α levels with preeclampsia are associated.^[39]^ In addition, immune cells, cytokines, ROS, and gastrointestinal microorganisms are involved in the pathophysiological processes of RA. The release of inflammatory diseases (such as TNF-α) and immunomodulatory factors (e.g., chronic inflammatory rheumatic diseases, gastroenterology, or skin diseases) may lead to the abnormal release of cytokines and chemokines in syncytial trophoblast cells. There is growing evidence that metabolic/pro-inflammatory cytokines can program placental function and growth in early gestation.^[40]^ In summary, RA during pregnancy can affect different molecular pathways associated with the pathogenesis of preeclampsia, such as angiogenesis, hypoxia, inflammatory signaling, thrombin or platelet activation, and vasoactive peptide imbalance. In conclusion, multiple mechanisms link RA during pregnancy to the subsequent development of vascular disease and preeclampsia.

## 5. Limitations

To our knowledge, this is the first meta-analysis describing the association between RA in pregnancy and the risk of preeclampsia. The main strength of our study is the use of a rigorous methodology for systematic evaluation and meta-analysis, which included an extensive literature search without language restrictions, risk of bias assessment in included studies, a quantitative summary of the evidence, and calculation of 95% prediction intervals. Additional strengths of our evaluation were the inclusion of a relatively large number of studies and women from diverse populations around the world. The results were robust when we performed sensitivity analyses using an exclusion method. In addition, there was no publication bias. Some potential limitations of our study should be considered. First, due to the low prevalence of preeclampsia, it is expected that the RRs and ORs produced were similar, and therefore both were combined as ORs. We pooled the unadjusted ORs (two studies) with the adjusted ORs (six studies), which may affect the interpretation of the results. And acknowledging that cohort studies and ORs often exaggerate effects, the pooled OR of 1.66 (with the upper limit of the CI of 1.80) is relatively modest (<2.0), so interpret this result with caution. Second, although subgroup analysis could explain some of the heterogeneity, there was a lack of sufficient primary data to investigate other potential sources, such as the characteristics of different types of RA (duration, severity, whether treated or not).

## 6. Conclusion

In conclusion, the evidence suggests pregnant women with RA are at higher risk for preeclampsia. While we recognize that confounding factors can affect the validity of the results, we were limited by the quality and scope of the included studies. Many of the studies did not report information on potential confounders, and we were, therefore, unable to control for these factors in our analysis. However, we conducted sensitivity analyses to evaluate the impact of potential confounders on our results and found that the overall conclusions of our meta-analysis remained robust. It will be necessary for future studies to report on potential confounding factors to improve the quality of evidence in this area. In clinical practice, monitoring of pregnant women with RA should be enhanced, and effective interventions such as low-dose aspirin use for pregnant women with RA should be considered to prevent adverse maternal and perinatal outcomes. Medical staff should develop an individualized and continuous pregnancy screening program for pregnant women with RA, record their family history, medical history, and physical examination data in detail, and provide health education on daily precautions for pregnant women. Before pregnancy, women with RA should be encouraged to maintain optimal health and manage their RA and other chronic diseases. Pregnant women with RA should schedule detailed and regular pregnancy tests according to the actual situation. By increasing the number of pregnancy tests, we can dynamically monitor the mother and fetus’s physiological changes and effectively reduce the risk of preeclampsia.

## Author contributions

**Conceptualization:** Lv Tian, Minru Zong.

**Data curation:** Lv Tian, Zhiyuan Zhang, Yuting Mao.

**Formal analysis:** Lv Tian, Zhiyuan Zhang, Yuting Mao.

**Funding acquisition:** Minru Zong.

**Investigation:** Lv Tian, Zhiyuan Zhang, Minru Zong.

**Methodology:** Lv Tian, Zhiyuan Zhang, Yuting Mao.

**Project administration:** Lv Tian, Zhiyuan Zhang, Minru Zong.

**Resources:** Minru Zong.

**Software:** Lv Tian, Zhiyuan Zhang, Yuting Mao.

**Supervision**: Minru Zong.

**Validation:** Lv Tian, Zhiyuan Zhang, Minru Zong.

**Visualization:** Lv Tian.

**Writing – original draft:** Lv Tian, Minru Zong.

**Writing – review & editing:** Lv Tian, Minru Zong.
